# How to Treat a Cyclist’s Nodule?—Introduction of a Novel, ICG-Assisted Approach

**DOI:** 10.3390/jcm13041124

**Published:** 2024-02-16

**Authors:** Julius M. Mayer, Sophie I. Spies, Carla K. Mayer, Cédric Zubler, Rafael Loucas, Thomas Holzbach

**Affiliations:** 1 Department of Plastic and Hand Surgery, Inselspital, University Hospital Bern, 3010 Bern, Switzerland; cedric.zubler@insel.ch; 2 Department of Dermatology and Allergy, Technical University Munich, 80802 Munich, Germany; 3 Department of Urology, Spital Thurgau, 8500 Frauenfeld, Switzerland; 4 Department of Hand and Plastic Surgery, Spital Thurgau, 8500 Frauenfeld, Switzerland; rafael.loucas@stgag.ch (R.L.); thomas.holzbach@stgag.ch (T.H.)

**Keywords:** cyclist’s nodule, perineal nodular induration, perineal tumor, third testicle, impaired lymphatic drainage, indocyanine green, ICG, plastic surgery, epicutaneous negative wound therapy, road cycling

## Abstract

Background: Perineal nodular induration (PNI) is a benign proliferation of the soft tissue in the perineal region that is associated with saddle sports, especially road cycling. The etiology has not been conclusively clarified; however, repeated microtrauma to the collagen and subcutaneous fat tissue by pressure, vibration and shear forces is considered a mechanical pathomechanism. In this context, chronic lymphedema resulting in the development of fibrous tissue has been suggested as an etiological pathway of PNI. The primary aim of this study was to introduce and elucidate a novel operative technique regarding PNI that is assisted by indocyanine green (ICG). In order to provide some context for this approach, we conducted a comprehensive review of the existing literature. This dual objective aimed to contribute to the existing body of knowledge while introducing an innovative surgical approach for managing PNI. Methods: We reviewed publications relating to PNI published between 1990 and 2023. In addition to the thorough review of the literature, we presented our novel surgical approach. We described how this elaborate approach for extensive cases of PNI involves surgical excision combined with tissue doubling and intraoperative ICG visualization for exact lymphatic vessel obliteration to minimize the risk of recurrence based on the presumed context of lymphatic congestion. Results: The literature research yielded 16 PubMed articles encompassing 23 cases of perineal nodular induration (PNI) or cyclist’s nodule. Of these, 9 cases involved females, and 14 involved males. Conservative treatment was documented in 7 cases (30%), while surgical approaches were reported in 16 cases (70%). Notably, a limited number of articles focused on histopathological or radiological characteristics, with a shortage of structured reviews on surgical treatment options. Only two articles provided detailed insights into surgical techniques. Similarly to the two cases of surgical intervention identified in the literature research, the post-operative recovery in our ICG assisted surgical approach was prompt, meaning a return to cycling was possible six weeks after surgery. At the end of the observation period (twelve months after surgery), regular scar formation and no signs of recurrence were seen. Conclusion: We hope that this article draws attention to the condition of PNI in times of increasing popularity of cycling as a sport. We aimed to contribute to the existing body of knowledge through our thorough review of the existing literature while introducing an innovative surgical approach for managing PNI. Due to the successful outcome, the combination of tissue doubling, intraoperative ICG visualization and postoperative negative wound therapy should be considered as a therapeutic strategy in cases of large PNI.

## 1. Introduction

Perineal nodular induration (PNI), more commonly known as “third testicle” or “cyclist’s nodule” [1], is a rare and benign (myo)fibroblastic pseudotumor associated with saddle sports, such as cycling or horseback-riding [2,3,4,5,6]. The clinical appearance of PNI is characterized by unspecific swelling of the soft tissue posterior at the perineum or over the ischial tuberosity [7]. The PNI manifests as two nodules, with one on each side of the perineal raphe; in other cases, it presents as a single nodule [1]. This localization of the swelling led to reports in journals on various medical disciplines, such as urology [2,8], gynecology [9,10], dermatology [1,3], orthopedic surgery [11] and plastic surgery, as well as histopathology [4,12] and radiology [13,14,15]. Overall, PNI is a rare disease and the knowledge about its treatment is limited. Between 1990–2023, a total of 16 PubMed listed articles involving 23 cases were published on the search term “perineal nodular induration or cyclist’s nodule”. Of these, 9 cases concerned female patients and 14 cases male patients. In 7 of the 23 (30%) cases, conservative treatment was described, while in 16 of the 23 cases (70%), surgical treatment approaches were discussed. Some of the 16 articles focused more on histopathological or radiological characteristics than on treatment options. To our knowledge, a structured review article on surgical treatment options is not yet available. Only two of these articles gave further insight into detailed surgical techniques. In one article, a spindle-shaped mass excision and wound closure by bilateral advancement flaps was mentioned [2]. Respectively, an incision at the lateral side of the perineum and a resection of the mass and simple wound closure was described in the other [16]. 

Histologically, a PNI is most likely the result of repeated microtrauma to the collagen and subcutaneous fat tissue by pressure, shear forces and vibration. Microtrauma can cause fibrinoid collagen degeneration with myxoid changes, pseudocysts formation and potential lymphatic vessel impairment [1]. Formation of a lymphatic drainage disorder can occur and result in chronic non-healing wounds. Although the etiology of PNI is not conclusively established, similarities in the clinical appearance to lymphedema have been observed. There is evidence in terms of similar appearance, continuous development with continued exposure and fibrotic remodeling during progression. For example, the altered skin with, in some cases, non-healing sores in advanced conditions resembles that of advanced chronic lymphedema skin alterations. One may suspect a relationship between the condition and a pressure/shear force-related chronic lymphatic drainage problem, and, indeed, in some histopathological examinations, obliterated and fibrosed lymphatic vessels were seen [9,17].

To the best of our knowledge, a proper classification of PNI has not yet been established. In our opinion, based on the size of the mass (small/medium/large in proportion to the total perineal area and the height of the tumor) and the patient’s discomfort, one might scale the PNI and decide on possible treatment options. For small lesions, conversative treatment is applicable. This includes the alteration of the cause, namely adjustment of the rider’s position on the saddle and wearing appropriate cycling pants. Rest alone was reported as not resulting in spontaneous regression of the mass [2,15]. However, further progression could be stopped by avoiding cycling. Complex physical decongestive therapy (CDT) is rarely mentioned in the context of PNI. Nevertheless, if PNI is pathogenetically related to lymphatic congestion, then conservative treatment strategies for lymphedema should be also considered for mild findings of PNI. The standard therapy for lymphedema is CDT, which consists of the following coordinated components: skin care and, if necessary, skin rehabilitation and manual lymphatic drainage, supplemented with additive manual techniques, compression therapy, education and instruction on self-treatment [18]. In a few case reports of small to medium PNIs, intralesional corticosteroid or hyaluronidase injections were found to offer some relief; however, these injections may cause subcutaneous atrophy [3,16,19,20]. These techniques are insufficient or, in some cases, even contraindicated in the setting of late-stage lymphedema and/or extensive tissue fibrosis (as late-stage PNIs), necessitating a more radical approach in terms of surgical excision. We describe our surgical treatment of extensive late-stage PNIs with a previously unreported technique based on the presumed context of lymphatic congestion using intraoperative ICG-monitoring for exact lymphatic vessel obliteration, which should minimize recurrence risk and allow for a prompt return to cycling. 

The clinical utilization of ICG has attained a well-established status in the field of plastic surgery. The ICG technique entails the intravenous or intradermal administration of a fluorescent solution, namely Verdye, in our hands (5 mg/mL, Diagnostic Green GmbH, München, Germany). This dye expeditiously forms complexes with plasma proteins and exhibits near-infrared fluorescence upon exposure to specific wavelengths. Within the realm of plastic surgery, surgeons employ specialized imaging devices to capture this fluorescence, facilitating real-time visualization of parameters such as blood flow, tissue perfusion and lymphatic drainage [21]. The dynamic information garnered through ICG administration significantly augments intraoperative decision-making capabilities, enabling the meticulous assessment of vascular integrity, identification of viable tissues and optimization of procedural outcomes, particularly in flap reconstructions and microvascular or lymphatic surgeries [22]. The technique’s non-invasive nature and swift clearance further bolster its safety profile and efficacy, underscoring its utility in guiding precise and sophisticated surgical interventions.

The aim of this article Is to contribute to the existing body of knowledge by providing a comprehensive review of the current literature while also introducing an innovative surgical approach for managing PNI. We hope that this article draws attention to the rather rare but also likely underdiagnosed condition of PNI [5], with special focus on the surgical treatment in cases of large PNI. 

## 2. Methods

Ethics approval: The study was approved by Spital Thurgau HPC Research Committee. No additional approval of the Kanthonal Ethics Board (EKOS Ostschweiz) was sought for the following reasons: outcome analysis was performed retrospectively on anonymised patient data, no additional examination of patients was planned nor performed, no potential change of treatment was implied. The study was performed in accordance with the ethical standards laid down in the 1964 Declaration of Helsinki and its later amendments.

The literature review was performed by including all PubMed listed articles referring to the search term perineal nodular induration or cyclist’s nodule published between 1990 and 2023. In particular, gender distribution and etiologic factors were reviewed, and the articles describing an operative procedure were analysed on surgical technique and outcome.

**Figure 1:**
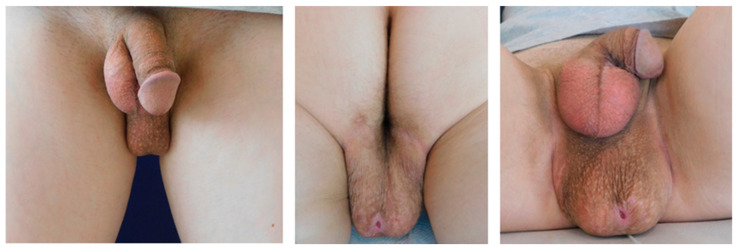
Clinical findings upon the first visit at our department of plastic surgery: The patient presented with a 12 × 6 × 4.5 cm large, mobile, non-tender, partially fibrotic mound of excess perineal tissue extending from the posterior base of the scrotum to the perianal area. On the base of the tumor, a small, circular, secreting ulceration could be seen that had only developed in the recent months.

**Figure 2:**
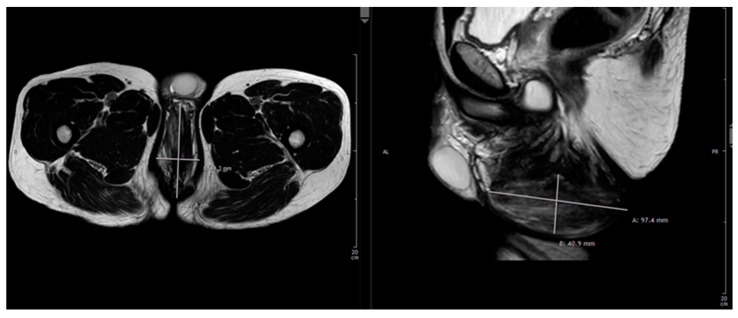
Diagnostic imaging: native and contrast enhanced T2-weighted MRI demonstrated a non-homogenous soft tissue proliferation with unclear demarcation to the surroundings, which was classified as a diffuse, partially fibrotic tissue transformation.

Presentation of our department’s experience in the context of PNI: A 42-year-old, otherwise healthy, male presented to our department of plastic surgery upon referral from the department of urology with a 10-year history with a nodular perineal lesion. He reported a progression of the swelling over the years, with the development of a small, non-healing, secreting wound becoming prominent in the past few months. At the time of presentation at our department, he was road cycling between 10–15 h weekly on a semi-professional basis and felt increasingly impaired by the tissue proliferation. The patient denied any sensibility impairment, erectile dysfunction or dysuria. Physical examination revealed a 12 × 6 × 4.5 cm large, mobile, non-tender, partially fibrotic mound of perineal tissue. On the base of the tumor there was a 3 mm small, sharply demarcated, circular secreting ulceration (see Figure 1). Ultrasonography revealed a mass of hypoechoic tissue with singular hyperechoic lesions and sparse perfusion inferior to the raphe, with no testicular abnormalities. Native and contrast enhanced T2-weighted MRIs demonstrated a non-homogenous soft tissue proliferation with unclear demarcation to the surroundings, which was classified as a diffuse, partially fibrotic tissue transformation (see Figure 2). The patient history and clinical findings pointed to the diagnosis of an extensive cyclist’s nodule with fibrosis related disruption of lymphatic drainage. Considering the available literature and the size of the lesion, we recommended surgical excision with tissue doubling and optional lymph vessel obliteration assisted by intraoperative ICG-monitoring in inpatient setting. 

**Figure 3:**
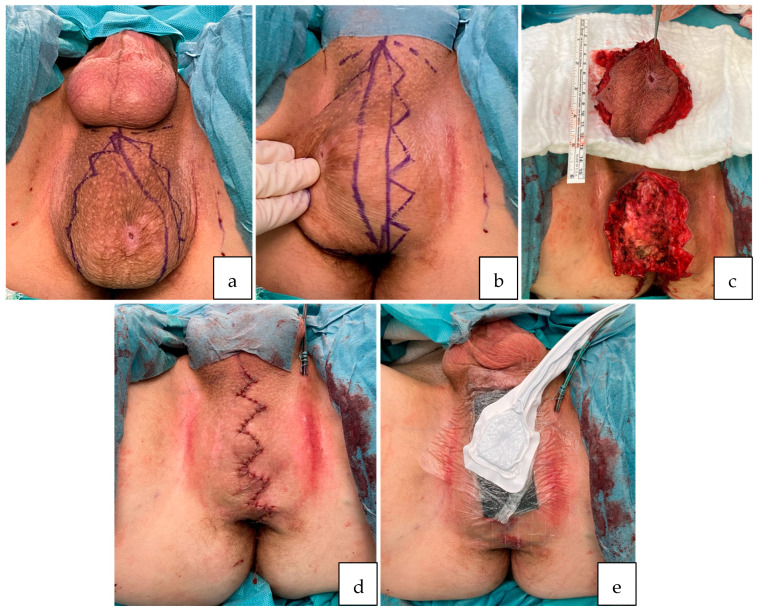
Step-by-step operative technique: (**a**,**b**) The patient was placed in a lithotomy position. The incision was marked, planning for a multiple W-plasty reaching from the apex, near the scrotum, down to 1 cm above the anus. (**c**) After deepithelialization of a 3 cm wide dermal strip to the left of the planned incision, the tumor was dissected en bloc with overlying tissue under conservation of blood vessels, nerves and surrounding muscle. (**d**) After hemostasis and obliteration of lymphatic leakages, a Redon drain (10 Charrière) was inserted before placing the deepithelialized strip underneath the opposing edge of the wound and fixing it transcutaneously to provide a padding-effect by doubling the tissue. The overlying W-plasty was adapted, and the skin was closed via simple interrupted suture. (**e**) A negative pressure wound therapy device was placed epicutaneously over the surgical wound, applying a continuous negative pressure of 125 mmHG.

**Figure 4:**
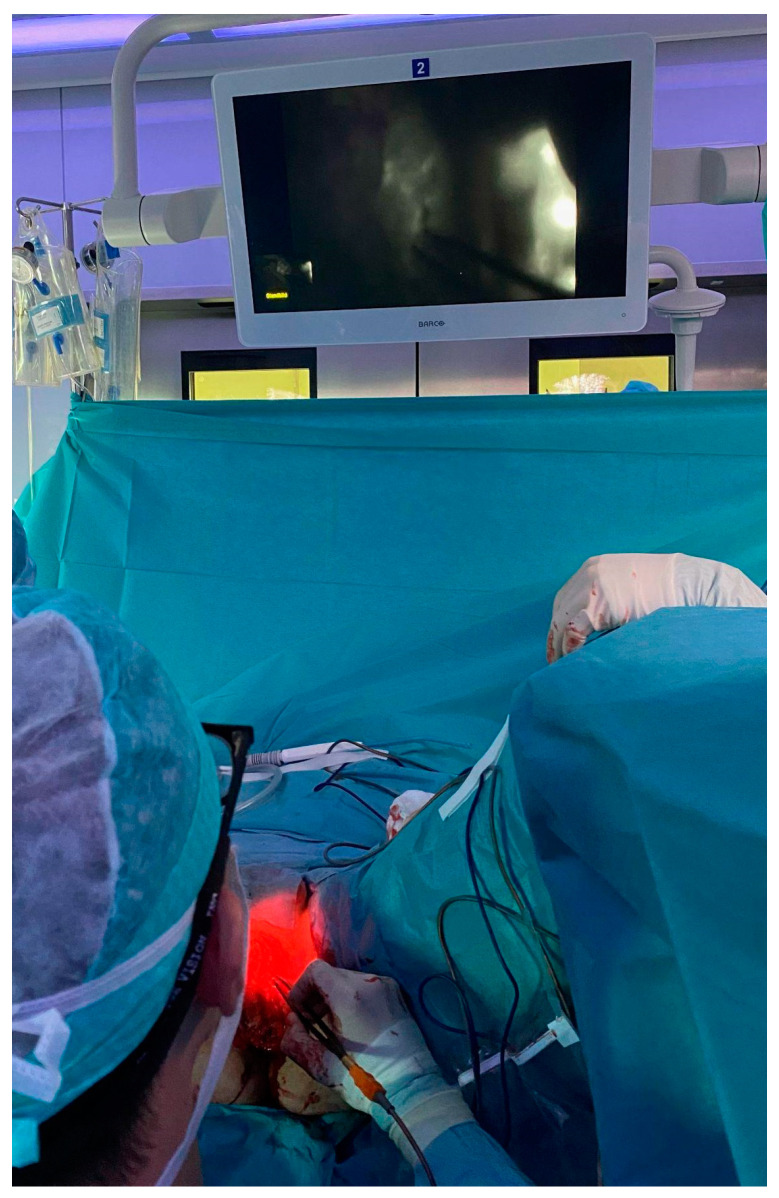
ICG imaging: 5 mL indocyanine green solution (VERDYE 5 mg/mL, Diagnostic Green GmbH, Germany) were injected subcutaneously bilaterally into the proximal inner thigh. An ICG handheld camera (IC-Flow™ Imaging System, Diagnostic Green GmbH, Germany) was used to detect lymphatic flow and possible leakages, which were subsequently obliterated using bipolar cautery forceps.

Surgical procedure: Surgery was performed under general anesthesia. The patient was placed in a lithotomy position. Perioperative antibiotic prophylaxis with amoxicillin/clavulanic acid was administered. The surgical procedure was performed under standard sterile conditions and surgical loupe magnification of ×2.5. The incision was marked, planning for a multiple W-plasty reaching from the apex, near the scrotum, down to 1 cm above the anus. Deepithelialization of a 3 cm wide dermal strip to the left of the planned incision was conducted before excision of the tumor. The tumor was dissected en bloc with overlying tissue under conservation of blood vessels, nerves and surrounding muscle (see Figure 3). The specimen was sent to the pathology department for histological examination. After this, 5 mL indocyanine green solution (VERDYE 5 mg/mL, Diagnostic Green GmbH, Germany) were subcutaneously injected bilateral into the proximal inner thigh for detection of lymphatic flow and possible leakages. With the help of the ICG handheld camera (IC-Flow™ Imaging System, Diagnostic Green GmbH, Germany), several small lymphatic vessels were identified and subsequently obliterated using bipolar cautery forceps (see Figure 4). Subtle thermic obliteration was performed for hemostasis before fitting a Redon drain (10 Charrière). The deepithelialized strip was placed underneath the opposing edge of the wound and fixed transcutaneously to provide a padding-effect by doubling the tissue. The overlying W-plasty was adapted and the skin closed via simple interrupted suture using Monocryl 4-0. Finally, a negative pressure wound therapy device was placed epicutaneously over the surgical wound, applying a continuous negative pressure of 125 mmHG. 

## 3. Results

The literature research yielded 16 PubMed articles encompassing 23 cases of perineal nodular induration (PNI) or cyclist’s nodule. Of these, 9 cases involved females, and 14 involved males. Conservative treatment was documented in 7 cases (30%), while surgical approaches were reported in 16 cases (70%). Notably, a limited number of articles focused on histopathological or radiological characteristics, with a shortage of structured reviews on surgical treatment options. Only two articles provided detailed insights into surgical techniques. 

Our patient’s recovery after the procedure was prompt and without complications. He mobilized on the next day and reported no pain under standard analgesic therapy with NSAIDs and paracetamol. The drain and vacuum dressing were removed upon dismissal two days after the surgery. The perioperative antibiotic prophylaxis with amoxicillin/clavulanic acid was continued for a total of five days after surgery. Early postoperative recommendations consisted of sexual abstinence, no bathing, and daily dressing changes for two weeks. 

Macroscopically the histopathological specimen consisted of skin and subcutaneous tissue, weighing 63 g. On the surface, there was a central, scarred ulcus measuring 1 × 1.2 cm. The cutting surface was inhomogeneous, white and soft with no clear demarcation of a focus border. Microscopically, the deeper tissues showed soft tissue collagen bundles mixed with fibroblasts, vessels and fat tissue. Focally, cystic degeneration was noted, but with no indication of a lymphocele or malignancy.

**Figure 5:**
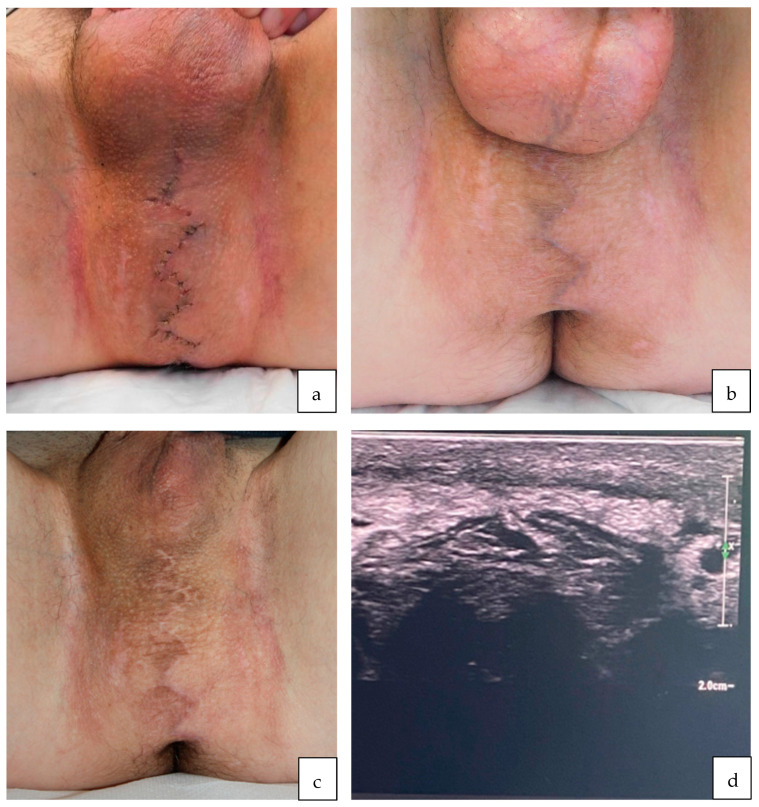
Clinical presentation of the surgical site at various follow-up visits: (**a**) Two weeks post-surgery showing regular wound healing, (**b**) six weeks post-surgery and (**c**) six months post-surgery showing minimal scar tissue formation (Vancouver scar scale) and no signs of a residual mass tissue. (**d**) The ultrasound at six months showed no sign of recurrence.

The patient was seen in our outpatient clinic electively one day after dismissal, and then two weeks, six weeks, six months and twelve months post-operatively. The wound healed uneventfully. At six weeks post-surgery, the patient reported reuptake of cycling without discomfort and still no issues with pain, sensibility impairment, erectile dysfunction or dysuria. Figure 5 shows the patient’s perineum and scrotum at the two week, six week and six month follow-up with regular wound healing, minimal scar tissue formation (Vancouver scar scale 0) and no signs of a residual mass tissue. Ultrasound examination six months post-surgery showed neither fluid collection in the sense of oedema nor signs of recurrence (Figure 5).

## 4. Discussion

Pathogenesis of PNI is attributed to the repetitive contact between the saddle and ischial tuberosities, and friction of the superficial perineal fascia against bony structures is thought to cause local injury, resulting in reparative proliferation of fibroblasts and myofibroblasts [3,4]. Chronic lymphedema resulting in the development of fibrous tissue has also been suggested as an etiological pathway of PNI [4]. Histopathological findings vary but tend to show necrosis of the superficial perineal fascia with myxoid degeneration of collagen fibers and fatty tissue, as well as occasional formation of a central pseudocyst [2,3,4,5,12,15]. In large PNIs, the skin condition with non-healing, exuding wounds often resembles other lymphatic problems, such as elephantiasis.

Clinical differential diagnoses vary in their likelihood depending on the patient’s age and sex. Common causes of perineal nodular lesions include hydrocoeles, varicoceles [23], lymph nodes, herniae, aneurysms [6] and various neoplasms, such as angiomyofibroblastoma (AMF)-like tumors and aggressive angiomyofibroblastoma [13,24]. Histopathologically, the central pseudocyst may resemble ischemic fasciitis, as seen in elderly patients with bony protuberances [2]. Due to the low number of published cases of PNI (namely the 23 PubMed listed between 1990 and 2023), it can be assumed that PNI is a relatively rare disease; however, no statement can be made regarding the absolute prevalence. Furthermore, the low number of published cases is also the reason why no significant difference in the gender-specific prevalence should be made, even though more case reports on male patients than on female patients have been published.

In most cases, typical patient history, localization, and appearance during physical examination can diagnose PNI. Additionally, imaging modalities, such as ultrasound and magnetic resonance imaging, can be used as non-invasive techniques to secure a diagnosis [13]. An algorithm published by Norman et al. in a case report in 2020 may also help reach a diagnosis in unclear cases [5].

In our hands, the diagnosis of PNI was formed based on the patient’s cycling history, clinical examination, as well as US and MRI imaging. 

Conservative treatment options that have been discussed include a reduction in cycling activity [3,6] and saddle adjustments, ranging from substituting a dome-shaped saddle with a flatter one to a cutout or channel through the middle of the saddle [5]. In cases where bike and saddle fit have already been maximized, and where a reduction in activity is unrealistic (such as in professional cyclists), intralesional corticosteroid or hyaluronidase injections may provide relief [3,16,19,20]. However, steroid injections are solely recommended for small to medium sized lesions due to the risk of subcutaneous atrophy [3]. 

Recently published cases of the results of conservative treatment of similar lesions have reported that the lesions remained stable for a period of 5 months [6] and 1 year [13] after a reduction in cycling activity and readjusting saddle fitting conditions.

However, a “steady state” is not in the interest of most patients due to the size of the lesion and the significant impairment it causes.

Surgical excision has been suggested as the treatment of choice in cases refractory to conservative treatment [2,16,19]. 

Then again, other authors have advised against the surgical removal of these lesions due the risk of recurrence and the potentially irritating nature of the resulting scar tissue [3]. Additionally, the role of lymphatic drainage in PNI also remains controversial. 

Based on our experience, we consider at least lymphatic impairment to be the main cause of the progression of a PNI. If, for example, a clinical picture of a PNI is compared with that of a penoscrotal lymphedema (PL), there is a striking similarity [24]. A recently published paper describes how, in advanced PL, surgical resection and penoscrotal reconstruction is the treatment of choice. In addition, Ehrl et al. suggest vascularized lymph node transfer (VLNT) to the groin or scrotum to be considered to improve the long-term surgical outcome in the sense of causal recurrence prophylaxis [25,26]. VLNT in general represents a surgical intervention for the management of lymphedeme. The procedure involves the extraction of healthy lymph nodes, typically sourced from the armpit or inguinal region, along with their associated vasculature. Employing precise microsurgical techniques, these nodes are intricately anastomosed to blood vessels at the targeted recipient site. Postoperatively, patients undergo a regimen of rehabilitation to optimize functional outcomes. Systematic follow-up assessments are imperative to evaluating the progression of the transplant’s efficacy. This nuanced surgical approach endeavors to ameliorate lymphatic drainage, mitigating edema and enhancing the overall quality of life [27]. In our surgical therapeutic approach to late-stage PNI, VLNT has not yet been an adjuvant treatment option, but may be discussed in cases of recurrence despite a previous elaborate surgical approach.

The clinical picture of the pronounced PNI clearly implied a lymphatic drainage problem. We decided to address the lymphedema/leakage issue using ICG-assisted intraoperative lymph vessel obliteration in addition to tissue doubling and W-plasty-designed incisions to prevent tension, as well as epicutaneous negative wound dressing.

The application of ICG is clinically well-established in the evaluation of tissue perfusion within the context of free flaps [21]. Furthermore, the ICG camera is used as an adjunctive method for the visualization of lymphatic flow and, lately, for the detection of sentinel lymph nodes [28,29]. ICG diagnostic agents involve non-ionizing radiation, and side-effects are rarely seen. The ICG handheld camera can help to identify lymphatic vessels intraoperatively, and it also allows the surgeon to obliterate lymphatic leakage upon detection. 

Due to the wound’s localization in an area where bacterial contamination from the anogenital tract is likely [30], we decided to protect it by using an epicutaneous negative wound therapy device for the first two postoperative days. The device reduces tension in the suture area and can prevent surgical site infections [11]. This kind of wound dressing proved to be comfortable both for the surgeon and the patient.

Comparing our surgical approach with alternative methods described in the literature, particularly in the context of PNI, is challenging due to the scarcity of studies directly comparing outcomes. Two other studies shed light on different surgical techniques for PNI, allowing for a preliminary exploration of comparative considerations.

Awad et al. presented a surgical excision approach involving a spindle-shaped mass excision and wound closure by bilateral advancement flaps [2]. In contrast, Peters et al. described an incision at the lateral side of the perineum, resection of the mass and simple wound closure [16]. These approaches provide valuable insights into the diversity of surgical strategies employed in managing PNI.

This article highlights the relevance to the various disciplines involved in the diagnostics and treatment of PNI, and it is the first to describe the use of intraoperative ICG visualization and the application of an epicutaneous negative wound therapy device in this context. This novel therapeutic strategy proved successful with the patient’s full return to health and no recurrence over the observation period of 12 months. 

## 5. Conclusions

Surgical excision combined with W-shaped tissue doubling, lymph vessel obliteration assisted by intraoperative ICG visualization and postoperative epicutaneous negative wound dressing proved to be a successful treatment strategy for large PNI. The application of ICG and epicutaneous negative therapy has not been described in the context of PNI treatment so far. Based on the presumed context of lymphatic congestion in PNI, we believe that ICG visualization can result in minimizing the risk of developing a postoperative lymphocele and the overall risk of recurrence.

However, it is essential to clarify that the conclusions drawn are primarily descriptive in nature. The uniqueness of our innovative surgical technique is presented, aiming to contribute to the evolving landscape of PNI treatment options.

Due to the limited availability of studies directly comparing outcomes across various surgical procedures and postoperative regimens for PNI, our discussion leans towards providing a detailed account of our approach rather than offering definitive conclusions regarding its superiority over alternative methods. 

Furthermore, we acknowledge the need for further research and comparative studies involving larger cohorts to comprehensively evaluate the efficacy and recurrence rates associated with different surgical interventions and postoperative care protocols. 

## Data Availability

Supporting data are available from the authors upon request.
